# Changes of dendritic cells and fractalkine in type 2 diabetic patients with unstable angina pectoris: a preliminary report

**DOI:** 10.1186/1475-2840-10-50

**Published:** 2011-06-10

**Authors:** Kang Yao, Hao Lu, Rongchong Huang, Shuning Zhang, Xiaowu Hong, Hongyu Shi, Aijun Sun, Juying Qian, Yunzeng Zou, Junbo Ge

**Affiliations:** 1 Department of Cardiology, Zhongshan Hospital, Shanghai Institute of Cardiovascular Diseases, 180 Fenglin Road, Shanghai China; 2 Institutes of Immunology, Fudan University,130 Dongan Road, Shanghai, China

**Keywords:** dendritic cell, fractalkine, unstable angina pectoris, type 2 diabetes, atherosclerosis, immune response

## Abstract

**Background:**

It has been shown that dendritic cells (DCs) and fractalkine play a role in accelerating progression of the inflamed atherosclerotic lesions and plaque rupture. We evaluated the numbers and functional changes of DCs and its subsets in human type 2 diabetes with or without unstable angina pectoris (UAP).

**Methods:**

The study population consisted of 39 diabetic patients (DM:18 without CAD; DM + UAP: 21 with UAP), 18 non-diabetic UAP patients (UAP), and 15 healthy control (Normal). Peripheral blood DCs and its subsets were measured by three color flow cytometry. Serum levels of fractalkine, IL-12, and IFN-α were also measured. The functional status of the monocyte-derived DCs was analyzed by flow cytometry and allogeneic mixed T lymphocytes reaction.

**Results:**

The percent and absolute numbers of DCs and mDC within the total leukocyte population was similar for Normal and DM, while significantly lower in DM + UAP. pDC numbers were not significantly altered. Serum fractalkine in DM + UAP was highest among the four groups (*p *= 0.04 vs. UAP, *p *= 0.0003 vs. DM, *p *< 0.0001 vs. Normal). Circulating mDC inversely correlated with serum fractalkine (r = -0.268, *p *= 0.01) level. Compared with DM and UAP, the costimulatory molecules CD86 and proliferation of T cells stimulated by DCs were significantly increased in DM + UAP group.

**Conclusions:**

Our study suggested that increases in the fractalkine level and the number and functional changes of blood DCs might contribute to diabetic coronary atherosclerosis and plaque destabilization.

## Introduction

Atherosclerosis and its clinic consequence, such as coronary artery disease (CAD), are the main cause of death in diabetic patients[1]. Coronary atherosclerotic plaques in diabetics tended to develop earlier and be more extensive and unstable compared with none diabetes[2,3]. Inflammation and immune reactions play a crucial role in the development of atherosclerosis and also diabetes associated vascular complications[4-6]. Immune responses induced by apoproteins and active moieties localized in the lipid fraction of the atheroma have been suggested to be involved in the inflammation response[7]. These antigens would attract antigen-presenting cells (APCs) and subsequent T cells activation in atherosclerotic plaque. Dendritic cells (DCs), the most potent APCs with the unique ability to initiate a primary immune response to certain antigens[8], were recently found to be markedly increased in atherosclerotic plaques[9-11] and were actively related in the process of atherosclerosis. Activated DCs regulate effector T cells which can kill plaque-resident cells and damage the plaque structure.

The chemokine family is thought to contribute significantly to the pathogenesis of atherosclerosis, from early plaque development to plaque rupture. The recently discovered chemokine fractalkine (FKN) and its receptor CX3CR1 are particularly interesting because of its potential pathophysiological role in atherosclerosis[12-14]. Fractalkine exists in two forms: the membrane-anchored multidomain protein which is expressed primarily on the surface of activated endothelial and smooth muscle cells, promoting the retention of monocytes and T cells, and the soluble form as a chemotactic stimulus, which strongly induces inflammation cells. It has recently shown that the level of FKN/CX3CR1 is increased in coronary artery disease, and may be related to plaque rupture[15,16]. In addition, a recent study[17] has shown that fractalkine is an important chemokine for accumulation of DCs in atherosclerotic plaque. Deficiency of the fractalkine receptor CX3CR1 resulted in decreased atherosclerosis and a decreased number of DCs in atheromas.

To the best of our knowledge, little is known about the expression of FKN in diabetic patients, and the role of DCs and FKN in human type 2 diabetes with or without CAD, particular in unstable coronary disease, remains unclear. We hypothesized that microenvironment in diabetes might result in changes of circulating DCs numeration and function, leading to the accelerated progression of diabetes-associated atherosclerosis. We have, therefore, characterized and compared circulating DCs subsets and FKN between type 2 diabetic patients with or without CAD and non diabetic controls. The maturation status and functional ability to stimulate T cell of DCs were also evaluated.

## Methods

### Subjects

The study population consisted of 39 diabetic patients with (DM + UAP, n = 21) or without (DM, n = 18) unstable angina pectoris (UAP) and 18 non-diabetic patients with UAP. They all underwent coronary angiography for the diagnosis of CAD in the Department of Cardiology, Zhongshan Hospital, Fudan University, Shanghai. UAP was defined as having ischemic chest pain at rest within the preceding 48 h, transient ST-T segment depression, angiographically verified at least one coronary lumen diameter narrowing > 50%, and no positive serum troponin I (> 0.04 ng/ml). The diagnosis of type 2 diabetes was made according to the criteria of the American Diabetes Association (ADA,2004). Patients who had acute or recent myocardial infarction, peripheral artery disease, type 1 diabetes, autoimmune diseases, malignancies, chronic or acute infections, asthma, severe heart failure (NYHA class 3 and 4) and advanced liver or renal diseases were excluded. In addition, 15 age and body mass index matched healthy subjects with neither diabetes mellitus nor any of the components of the metabolic syndrome were studied as Normal group.

Blood were obtained into EDTA tubes from all subjects via antecubital venepuncture soon after admission. The protocol was approved by the hospital Ethics Committee and written informed consent was obtained from all patients. The study was performed according to the principles of the Declaration of Helsinki.

### Biochemical analysis

White blood cell, serum cholesterol, triglycerides and glucose were measured according to routine protocols. Serum HbA1c was determined by HPLC using the BioRad Variant System. The serum level of FKN was analyzed using enzyme-linked immunosorbent assay kits (DY365 DuoSet System, R&D Systems) according to the manufacturer's instructions. The serum level of IL-12 and IFN-α were assayed by ELISA (Bender MedSystems, Austria).

### Monoclonal antibodies

Phycoerythrin (PE)-conjugated CD11c, PE-conjugated anti-IL-3 receptor α chain (CD123), peridinin chlorophyll protein (PerCP)-conjugated anti-HLA-DR, fluorescent isothiocyanate (FITC)-conjugated lineage cocktail 1 (lin1) and CD86-FITC were purchased from Becton Dickinson (San Jose, CA, USA). The lin1 contains monoclonal antibodies (mAbs): CD3 (T cells), CD14 (monocytes/macrophages), CD16 (natural killer cells), CD19 (B cells), and CD56 (natural killer cells). PE-conjugated isotype control murine mAbs were obtained from Becton Dickinson.

### Enumeration of peripheral blood DC

**Figure 1:**
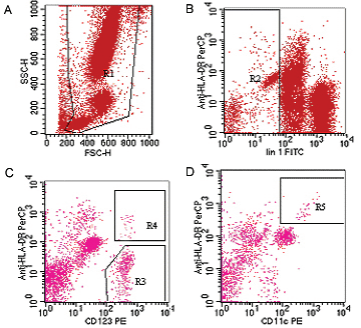
**Detection of fractions of peripheral blood dendritic cells (DCs) by standardized 3-color flow cytometry**. Peripheral blood samples were collected and the cells were stained with PerCP-conjugated anti-HLA-DR, PE-conjugated anti-CD11c or anti-IL3α (CD123), and FITC-conjugated lineage markers (lin1) containing anti-CD3, CD15, CD16, CD19, CD20, and CD56. After lysis of erythrocytes, the cells were analyzed by three-color flow cytometry. A: Events excluding debris and dead cells (R1); B: cells were gated on region R1, a dot blot of lineage marker (x axis) versus HLA-DR (y axis) was used to define the region of Lin^-^cells (R2); (C) pDC was defined as Lin1^-^, HLA-DR ^+ ^, and CD123 ^+ ^(R4); (D) mDC was defined as Lin1^-^, HLA-DR ^+ ^, and CD11c ^+ ^(R5). FITC: fluorescein isothiocyanate; FSC-H: forward scatter; PE: phycoerythrin; mDC: myeloid dendritic cell; pDC plasmacytoid dendritic cell.

Peripheral blood cells, obtained from the subjects in a prospective manner, were analyzed by three-color flow cytometry as we published previously[18]. Briefly, 200 μl blood was incubated with PE-, PerCP-, and FITC-conjugated mAbs for 20 min at room temperature. The erythrocytes were then lysed with FACS lysing solution (Becton Dickinson). After washing with FACS flow, the stained cells were analyzed with a FACS Caliber flow cytometer and the CellQuest Pro software (Becton Dickinson). To gate Lin1- HLA-DR + cells, whole peripheral blood cells were stained with anti-HLA-DR mAb and the Lineage Cocktail (Figure 1). In the gated cells we further defined the expression of CD11c and CD123 to determine the two distinct DC lineages. mDC and pDC were defined as Lin1^-^HLA-DR ^+ ^CD11c ^+ ^and Lin1^- ^HLA-DR ^+ ^CD123 ^+ ^, respectively (Figure 1). The number of total white blood cells in the samples was determined using an automated cell counter. CD11c or CD123 conjugated with PE was used for identification of the mDC and pDC subsets. Absolute numbers of mDC and pDC were calculated from the white blood cell count multiplied by the proportion of each subset within the white blood cells. The percentage of mDC and pDC was derived from the total number of DCs as determined by flow cytometric analysis.

### Generation of Dendritic Cells from Monocytes

We used monocyte-derived DCs to access their maturation and functional activation status. Twenty ml of blood each was collected from subjects of the four groups. Isolation of peripheral blood mononuclear cells and generation of dendritic cell was performed as we described before[19]. Briefly, blood was diluted 1:2 in PBS layered over Histopaque 1077 (Sigma, USA) and centrifuged for 30 min at 2000 rpm at room temperature. The interface was recovered and washed three times in PBS. CD14 ^+ ^PBMC were purified by using CD14 ^+ ^immunomagnetic micro beads (Miltenyi Biotech GmbH, Germany) and incubated in RPMI-1640 medium supplemented with GM-CSF (100 ng/mL) and IL-4 (50 ng/mL) in six-well tissue culture plates at 37°C and an atmosphere of 5% CO_2_. The medium was replaced every 2 days. After seven days, the cultures developed an adherent monolayer and clusters of DC colonies.

### Evaluation of maturation state of DCs

We examined the expression level of CD86 to access the maturation state of DCs. DCs(1 × 10^6 ^cells) were harvest and blocked with 10% normal goat serum for 15 min at 4°C, washed and then stained with FITC-conjugated antibodies against CD86 for 30 min at 4°C. Cells stained with the appropriate isotype-matched IgG were used as negative controls. After immunofluorescence staining, cells were analyzed by FACS Calibur using CellQuest software.

### Dendritic cell-induced T cell response

For the mixed lymphocyte reaction, T lymphocytes were isolated from peripheral blood by human T cell recovery immunocolumn kit (Cedarlane Laboratories Ltd, Canada). MLRs were conducted in 96-well flat-bottom culture plates. Before mixed, DCs was pretreated by mitomycin (150 μmol/L) for 1 hour at 37°C. These cells were then cultured with 2 × 10^5 ^T cells in 200 μL complete culture medium at 1:5, 1:10, 1:50 and 1:100 DCs:T cells ratio. After 96 hours, 50 μL culture supernatants were collected, stored at -70°C, and analyzed by ELISA for production of IL-2, a marker of T-cell activation. Then 50 μL fresh medium containing 1 μCi of [^3^H] thymidine were added. After 16 hours, the cells were harvested onto filter paper, and the incorporation of [^3^H] thymidine was determined by scintillation counting.

### Statistical analysis

All statistical analyses were performed using SPSS for Windows 13.0 (SPSS Inc., Chicago, Illinois). Data are presented as frequencies and percentages for categorical variables and mean ± SD for continuous variables, unless otherwise indicated. Statistical analyses were performed with the unpaired Student t test/Mann-Whitney U test or one-way ANOVA when indicated and Chi square test to compare the differences of two groups. The correlation between variables was analyzed by linear regression analysis. The p values less than 0.05 were considered to indicate statistical significance.

## Results

### Study population and clinical characteristics

There was no statistical difference among four groups with regard to age, gender, BMI, HDL-C and white blood counts. Triglyceride, total and LDL-cholesterol levels in the DM, UAP and DM + UAP are higher than that of the Normal. There was no statistical difference between the two groups of DM and DM + UAP in terms of duration of diabetes, fasting blood glucose and serum HbA1c. Medication using among the groups of DM, UAP and DM + UAP was similar before admitting and blood taken, particularly in the use of anti-platelet therapy, angiotensin-converting enzyme antagonists, or statins. The demographic details of the populations studied are given in Table 1.

**Table 1:** Study population and clinical characteristics

	Normal (n = 15)	DM (n = 18)	UAP (n = 18)	DM + UAP (n = 21)
Age(y)	54.2 ± 11.4	56.2 ± 8.2	57.5 ± 10.5	55.3 ± 11.2
BMI(kg/m^2^)	23.7 ± 1.3	24.2 ± 5.3	25.1 ± 3.6	24.6 ± 4.1
Hypertension(n)	0	12*	14*	18*
Diabetes duration(y)	---	3.9 ± 1.0	---	4.1 ± 0.8
TC(mmol/l)	3.1 ± 0.5	4.6 ± 1.4*	4.7 ± 2.0*	4.9 ± 1.8*
LDL-C(mmol/l)	1.6 ± 0.4	2.9 ± 0.7*	3.1 ± 0.8*	3.0 ± 1.5*
HDL-C (mmol/l)	1.2 ± 0.5	1.2 ± 0.7	1.1 ± 0.8	1.2 ± 0.6
TG (mmol/l)	1.3 ± 0.7	2.2 ± 1.8*	1.9 ± 1.5*	2.0 ± 1.6*
WBC(× 10^9^)	5.9 ± 0.9	7.2 ± 0.8	7.5 ± 0.8	6.8 ± 1.0
Fasting glycaemia(mg/ml)	4.6 ± 0.7	8.5 ± 2.3^#^*	5.2 ± 1.2	8.1 ± 1.7^#^*
HbA1c(%)	4.4 ± 0.88	8.4 ± 2.5^#^*	5.1 ± 1.1	7.8 ± 1.8^#^*
*Medications*				
Statins	0	16	18	20
Aspirin	0	16	18	21
Fibrates	0	1	0	1
ACE inhibitor/ARB	0	13	14	17

Results are expressed as mean ± SD. Normal: normal control subjects; DM: type 2 diabetic patients without CAD; UAP: non-diabetic patients with unstable angina pectoris; DM + UAP: type 2 diabetic patients with UAP; ACE: angiotensin-converting enzyme; ARB: angiotensin-receptor blocker. *p < 0.05 versus Normal; ^#^*p *< 0.05 versus UAP.

### Concentration of Circulating sFKN and Other Markers of Inflammation

The results showed that mean serum FKN level was 477 ± 189 pg/ml in the normal subjects, 572 ± 165 pg/ml in diabetic patients without CHD, 729 ± 145 pg/ml in non-diabetic patients with UAP, and 943 ± 365 pg/ml in diabetic patients with UAP. Mean FKN level of DM + UAP group was not only higher than the group of UAP (*p *= 0.04), but also than that of DM group(*p *= 0.0003). It was also higher in UAP than DM group (*p = *0.03). On the contrary, mean FKN level of DM was not different from the Normal.

Serum IL-12 levels were significantly higher in the DM, UAP and DM + UAP groups than in the Normal group (Table 2), and were significantly higher in the DM + UAP group than in the DM and UAP groups(p = 0.01 and 0.001 respectively). On the other hand, there were no significant differences in the serum interferon-α level among the 4 groups (Table 2).

**Table 2:** Concentration of circulating sFKN, IL-12 and IFN-α

Molecules	Normal (n = 15)	DM (n = 18)	UAP (n = 18)	DM + UAP (n = 21)
*sFKN, pg/ml*	477.1 ± 189.4	572.2 ± 165.0	729.0 ± 245.4*^#^	942.6 ± 364.6*^#†^
*IL-12, pg/ml*	45.7 ± 15.7	85.4 ± 35.6*	74.2 ± 32.4*	119.5 ± 44.1*^#†^
*IFN-α, pg/ml*	3.3 ± 3.7	2.0 ± 1.8	3.1 ± 2.7	2.9 ± 3.2

Results are expressed as mean ± SD. Normal: normal control subjects; DM: type 2 diabetic patients without CAD; UAP: non-diabetic patients with unstable angina pectoris; DM + UAP: type 2 diabetic patients with UAP. *p < 0.05 versus Normal; ^#^*p *< 0.05 versus DM; †*p *< 0.05 versus UAP.

### Frequency of circulating total DCs and DCs subsets

**Figure 2:**
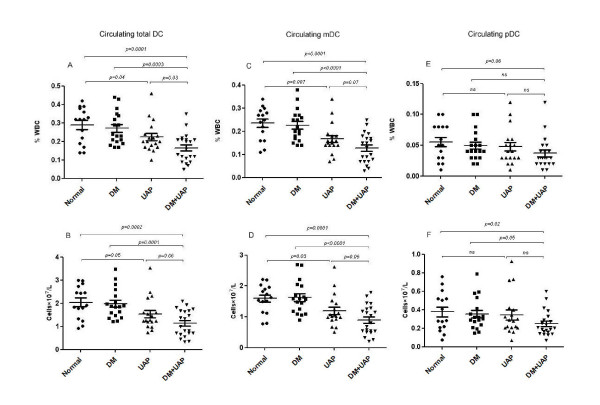
**Percentage and absolute numbers of peripheral blood total DCs (A,B), mDC(C,D) and pDC (E,F) in normal control (Normal, n = 15), type 2 diabetic patients with (DM + UAP, n = 21) or without unstable angina pectoris(UAP) (DM, n = 18), and non-diabetic UAP patients(UAP, n = 18)**. Horizontal long lines mean values. mDC: myeloid dendritic cell; pDC plasmacytoid dendritic cell.

The percent and absolute number of DCs within the total leukocyte population was similar in type 2 diabetic patients without CAD (0.27 ± 0.09% and 1.98 ± 0.64 × 10^7^/L) and the Normal controls (0.29 ± 0.10% and 2.04 ± 0.71 × 10^7^/L). Also no differences were found in the number of mDC and pDC between the two groups (mDC, 0.23 ± 0.07% and 1.62 ± 0.53 × 10^7^/L versus 0.24 ± 0.07% and 1.59 ± 0.46 × 10^7^/L; pDC, 0.05 ± 0.02% and 0.35 ± 0.17 × 10^7^/L versus 0.06 ± 0.03 and 0.38 ± 0.21 × 10^7^/L) (Figure 2). The ratio of mDC:pDC was also unchanged with mDC comprising the vast majority of DCs.

However, the percentage and absolute number of total DCs was significantly lower in diabetic patients with UAP compared to those without CAD (0.17 ± 0.07 versus 0.27 ± 0.09%, p = 0.0003; 1.14 ± 0.57 versus 1.98 ± 0.64 × 10^7^/L, p = 0.0001). Moreover, mDC levels were also significantly decreased in diabetic patients with UAP compared to that of without CAD, when analyzed as percentage of total white cells (0.13 ± 0.06% vs. 0.23 ± 0.07%, p < 0.0001) or as absolute DCs (0.90 ± 0.48 × 10^7^/L vs. 1.63 ± 0.53 × 10^7^/L, p < 0.0001). In contrast, pDC numbers were not significantly altered (0.04 ± 0.03% versus 0.05 ± 0.02%, p > 0.05) between the two groups. While compared with Normal the number of absolute pDC was significantly decreased in diabetic patients with UAP (p = 0.02) (Figure 2).

The results also showed that percentage of total DCs was significantly lower in diabetic patients with UAP compared with non-diabetic UAP patients (0.17 ± 0.07 versus 0.22 ± 0.08%, p = 0.03). We also found the significant decrease of DCs and mDC in patients of UAP compared with the Normal control subjects, which had been reported before.

### Assessment of the maturation of DC in the different groups

**Figure 3:**
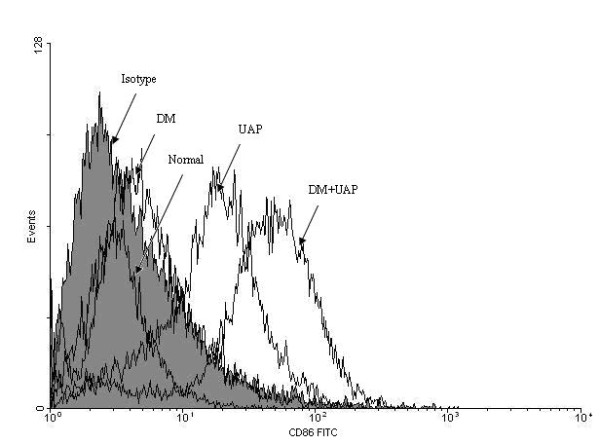
**The immunophenotypic expressions of CD86 on monocyte-derived DCs in subjects of Normal, DM, UAP and DM + UAP**. Flow cytometric analysis was performed for surface CD86 expression examination. Normal: normal control subjects; DM: type 2 diabetic patients without CAD; UAP: non-diabetic patients with unstable angina pectoris; DM + UAP: type 2 diabetic patients with UAP

The expressions of surface CD86 was marker that DCs grew from immature state to maturation. Fluorescence-activated cell sorter (FACS) analysis showed that the expression of CD86 in patients with DM + UAP was the highest among the four groups. It was also significantly increased in patients with DM + UAP compared with UAP. Differences between patients with DM and Normal were not significant (Figure 3). It indicated that the state of DCs in patients with DM + UAP was more mature than that in diabetic patients with no CAD and normal control.

### The capacity of DCs to stimulate T cells in the different groups

**Figure 4:**
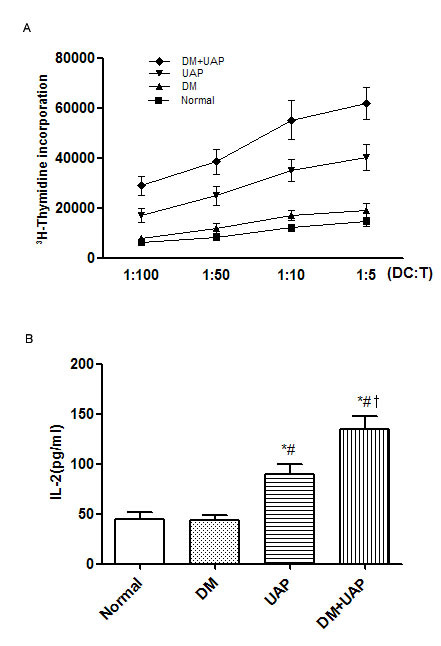
**Stimulating effects of monocyte-derived DCs on T cells in MLRs**. A: The incorporation of [^3^H] thymidine was determinated by scintillation counting at 1:5, 1:10, 1:50 and 1:100 DCs:T cells ratio. B: The amounts of IL-2 in the supernatants of the culture at 1:5 ratios was measured by ELISA. *P < 0.05 vs. Normal; ^#^*P *< 0.05 vs. DM; †*P *< 0.05 vs. UAP. MLR:mixed T lymphocytes reaction

To evaluate the functional activity, we next examined the capacity of DCs to stimulate T cells in the different groups by use of allogeneic MLRs. Compared with DM and UAP, the proliferation of T cells stimulated by DCs in DM + UAP was significantly increased. The capacity of DCs in UAP was also significantly higher than that of DM and Normal, and differences between patients with DM and controls were not significant.(Figure 4A). Simultaneously, DM + UAP had markedly increased T-cell secretion level for IL-2 compared with DM (by more than 3-fold at 1:5 DCs/T cells ratio) and UAP(p = 0.01), consistent with T-cell activation.(Figure 4B).

### Association of circulating mDC with inflammation and atherogenic markers

**Figure 5:**
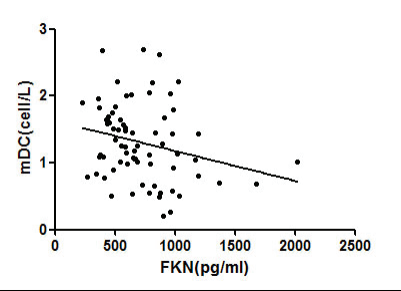
**A significant inverse correlation of circulating mDC with serum levels of fractalkine**. mDC: myeloid dendritic cell.

Circulating mDC inversely correlated with serum FKN(r = -0.268, p = 0.01) (Figure 5). No statistically significant correlation was found between mDC and IL-12 level.

## Discussion

The main findings in this study are the quantitative and functional abnormalities observed in circulating DCs detected in the diabetic patients with coronary artery disease. Our study found that the number of circulating total DCs and mDC in type 2 diabetic patients with UAP decreased significantly compared with that without CAD and nondiabetic patients with UAP, while pDC was not significantly altered. Furthermore, functional status of DCs in diabetic patients with UAP were more mature and activated than diabetic patients without CAD and none diabetic patients with UAP. This was evidenced by the upregulation of costimulatory molecule CD86 and the enhanced capability of DCs to stimulate T-cell proliferation and cytokine production in allogeneic MLRs. Our data also showed FKN, an important chemokine for accumulation of DC in atherosclerotic plaque, was significantly increased in diabetic patients with UAP compared with those without CAD, and the decline in mDC inversely correlated with the level of sFKN.

Diabetes is a widely accepted risk factor for atherosclerosis. Insulin resistance and hyperglycemia in type 2 diabetes mellitus are associated with a systemic proinflammatory state that promotes the development of atherosclerosis[20,21], as evidenced by increases in the concentration of proinflammatory cytokines, such as IL-6, which plays a deleterious role in the development of CAD and is an early indicator of incipient type 2 diabetes mellitus. The inflammatory response was greater in diabetic than in nondiabetic plaques. Our recent in vitro studies have reported that advanced glycosylation end products (AGEs) [19], hyperinsulin[22] could enhance DCs maturation and induce an antigen-specific T cell activation. The present study in diabetic patients further supported the role of DCs and its subsets on the immune reactions in diabetic atherosclerosis and plaque destabilization. CD86 is one of the mature markers of DCs. Costimulation by the ligand CD86 and its receptor CD28 on T cells is required for efficient T-cell stimulation. We detected the expression level of CD86 in DCs in different groups. We found that the expression level of CD86 in DM + UAP patients was significantly higher than in DM and UAP groups. Furthermore, co-culture with DCs from DM + UAP patients evoked strong proliferation of the T cells in comparison with the groups of DM, UAP and Normal. DCs maturation and T-cell stimulation in DM + CAD groups was paralled with more IL-2 secretion, which is a marker of T-cell activation. Considering that DCs are most frequently observed in atherosclerotic lesions enriched with T cells, the increased expression of costimulatory molecules on DCs and the capacity of T cell stimulation as well as cytokine secretion seen in DM + UAP patients suggest that activated function of DCs in these patients is an important mediator in immuno-inflammatory process as they are responsible for T-cell activation and this cellular interaction plays a role in plaque instability and vulnerability towards rupture.

There are several possible reasons for the decline of blood DCs in diabetic patients with unstable coronary artery disease. Firstly, the inflammatory response was greater in diabetic than in nondiabetic plaques and circulating DCs were trafficked into inflamed vulnerable plaques. Indeed, it has been reported that atherosclerotic arteries and vulnerable plaques contains more DCs than unaffected arteries[10,23,24]. Yilmaz A et al[11] recently described a significant decrease in circulating mDC precursors in patients with acute coronary syndromes, and this is associated with obvious accumulation in mDC in vulnerable atherosclerotic plaques. The observed more profound decrease in mDC than in pDC might result from differences of their function and the migratory response to chemotactic stimuli. The hypothesis is supported by our data that compared with diabetes without UAP, patients with diabetic unstable coronary disease have a significantly higher level of FKN, which has been shown to be an important chemokine for mDCs. pDC respond only to the homeostatic CXCL-12, which attracts them into lymphoid organs[25]. Stimuli known to accelerate atherosclerosis in diabetes, such as oxidized LDLs[26], AGEs[19], hypoxia[27]could role as antigens to induce DCs to adhesion to and transmigration through endothelial cells. Because mDC and pDC are considered to be specialized APCs preferentially inducing a Th1 and Th2 response respectively[28], the migration of circulating mDC into atherosclerotic plaque might play an essential role in the initiation and regulation of local immune reactions in plaque by polarizing naïve T-helper cells into Th1 effector cells, thus enhancing a Th1 response. Indeed, despite the decrease in circulating mDC, we found that serum IL-12 levels which is a Th1 type cytokine and mainly secreted by mDCs was significantly higher in the DM + UAP group compared with those in the DM, UAP groups and Normal controls, suggesting that mDCs in peripheral blood are activated to secrete cytokines in diabetic patients with UAP. Secondly, we can not exclude the possibility that DCs after engulfing antigen in the arterial wall migrated into regional lymph nodes where they activate T cells. Supporting this possibility are the immunohistochemical observations showing that the number of DCs in lymph nodes attached to atherosclerotic wall segments exceed those in lymph nodes attached to nonatherosclerotic arteries[29]. Thirdly, reduced generation of DCs in bone marrow, disturbed differentiation of blood DCs from CD34 ^+ ^precursors, might also explain the decreased blood DCs numbers in diabetic patients with CAD. Indeed, for example, although TNF-α is required for DCs generation from CD34 ^+ ^precursors, its increased production by monocytes in type 2 diabetes with CAD may adversely affect blood DC differentiation from CD34 ^+ ^precursors[30,31]. In addition, the decreased DCs counts do not seem to be related to suppressed myelopoiesis since patients of in type 2 diabetes with CAD in our study had similar monocyte counts to those without CAD and controls.

Taken together, our study showed a significant decrease in circulating total DCs and mDC in type 2 diabetic patients with UAP, and functional status of DCs in diabetic patients with UAP were more mature and activated than diabetic patients without CAD and none diabetic patients with UAP. The decline in mDC inversely correlated with the serum level of FKN which has been shown to be an important chemokine for accumulation of DCs in the atherosclerotic plaque. Because DCs are most frequently observed in atherosclerotic lesions enriched with T cells and are strong T-cell stimulators, our results suggest that the number and functional changes of DCs in diabetic patients with UAP might plays an important role in this cellular interaction, leading to plaque instability and vulnerability towards rupture. Because of the limitation in small population of our study, furthered detailed studies with larger cohort will no doubt lead to improved knowledge regarding the role DCs play in diabetic atherosclerotic lesions. Moreover, monitoring of DCs and its subsets may represent a novel strategy to detect acute coronary syndrome in human type 2 diabetes.

## Competing interests

The authors declare that they have no competing interests.

## Abbreviations

CAD: coronary artery disease; APCs: antigen-presenting cells; DCs: dendritic cells; mDC: mysloid dendritic cell; pDC: plasmacytoid dendritic cell;sFKN: serum fractalkine; UAP: unstable angina pectoris; DM diabetes mellitus;AGEs: advanced glycosylation end products;MLR: mixed T lymphocytes reaction; FITC: fluorescein isothiocyanate; FSC-H: forward scatter; PE: phycoerythrin; IgG: immunoglobulin G; GM-CSF: granulocyte-macrophage colony-stimulating factor;IL-4: interleukin-4; IL-12: interleukin-12;IFN-α: interferon-alpha;CX3CR1:C-X3-C motif receptor 1.

## Authors' contributions

KY: study design and perform, data analysis, manuscript preparation; HL: study perform, data analysis and discussion, manuscript editing;RCH: study perform, manuscript editing; SNZ: data acquisition, statistical analysis, manuscript editing; XWH: study design, manuscript editing; HYS: data analysis, manuscript editing; AJS: final approval of the manuscript; JYQ: study design, final approval of the manuscript; YZZ: study design, final approval of the manuscript, JBG: study design, manuscript editing, final approval of the manuscript; manuscript editing, KGP: study design, manuscript editing. All authors read and approved the final manuscript.
